# Sodium butyrate inhibits aerobic glycolysis of hepatocellular carcinoma cells via the c‐myc/hexokinase 2 pathway

**DOI:** 10.1111/jcmm.17322

**Published:** 2022-04-16

**Authors:** Qiang Yu, Weiqi Dai, Jie Ji, Liwei Wu, Jiao Feng, Jingjing Li, Yuanyuan Zheng, Yan Li, Ziqi Cheng, Jie Zhang, Jianye Wu, Xuanfu Xu, Chuanyong Guo

**Affiliations:** ^1^ Department of Gastroenterology Shanghai Tenth People’s Hospital School of medicine, Tongji University 200072 Shanghai China; ^2^ Department of Gastroenterology Shidong Hospital, Yangpu District Shidong Hospital Affiliated to University of Shanghai for Science and Technology 200433 Shanghai P.R.China; ^3^ Department of Gastroenterology Putuo People's Hospital Tongji University 200060 Shanghai China

**Keywords:** aerobic glycolysis, hepatocellular carcinoma, hexokinase 2, sodium butyrate, sorafenib

## Abstract

Aerobic glycolysis is a well‐known hallmark of hepatocellular carcinoma (HCC). Hence, targeting the key enzymes of this pathway is considered a novel approach to HCC treatment. The effects of sodium butyrate (NaBu), a sodium salt of the short‐chain fatty acid butyrate, on aerobic glycolysis in HCC cells and the underlying mechanism are unknown. In the present study, data obtained from cell lines with mouse xenograft model revealed that NaBu inhibited aerobic glycolysis in the HCC cells *in vivo* and *in vitro*. NaBu induced apoptosis while inhibiting the proliferation of the HCC cells *in vivo* and *in vitro*. Furthermore, the compound inhibited the release of lactate and glucose consumption in the HCC cells *in vitro* and inhibited the production of lactate *in vivo*. The modulatory effects of NaBu on glycolysis, proliferation and apoptosis were related to its modulation of hexokinase 2 (HK2). NaBu downregulated HK2 expression via c‐myc signalling. The upregulation of glycolysis in the HCC cells induced by sorafenib was impeded by NaBu, thereby enhancing the anti‐HCC effect of sorafenib in *vitro* and *in vivo*. Thus, NaBu inhibits the expression of HK2 to downregulate aerobic glycolysis and the proliferation of HCC cells and induces their apoptosis via the c‐myc pathway.

## 1 INTRODUCTION

Primary liver cancer is responsible for over 8 million deaths worldwide annually and is the sixth most frequently diagnosed cancer and the third leading cause of cancer‐related death, which makes it a serious global health burden.^1^ Hepatocellular carcinoma (HCC) accounts for 75%–85% of cases of primary liver cancer. Thus, finding a safe and effective strategy to treat patients with HCC is urgently needed.^2^

In 2011, energy metabolism reprogramming was identified to be a novel hallmark of cancer.^3^ In 1930, Otto Warburg found that in the presence of sufficient oxygen, cancer cells tended to convert glucose to lactate via glycolysis instead of catabolizing it via oxidative phosphorylation (OXPHOS), a phenomenon termed ‘aerobic glycolysis’ or ‘Warburg effect’.^3^ Enhanced aerobic glycolysis was observed in HCC,^4^ lung cancer,^5^ breast cancer^6^ and other types of cancer. High‐speed energy production, synthesis of metabolic substrates and lactate production via aerobic glycolysis promote hepatocellular carcinogenesis by regulating the proliferation, growth, immune evasion, invasion, metastasis, angiogenesis and sorafenib resistance of HCC.^7^ Moreover, a recent review indicated that key enzymes in the glycolytic pathway modulate several important phenotypes of cancer, including DNA repair, mitosis, regulation of exosomes and ectosomes, autophagy, and mitochondria‐dependent apoptosis, via noncanonical functions.^8^ Previous experimental studies found that glycolysis inhibitors, such as aspirin,^9^ simvastatin^10^ and shikonin,^11^ could exert anti‐HCC effects and enhance the sensitivity of the HCC cells to sorafenib, an oral multikinase inhibitor, in the treatment of advanced HCC. Hence, targeting the glycolytic pathway and its key enzymes could exert antitumour effects by regulating diverse key phenotypes of cancer and has become a novel strategy in HCC treatment. Moreover, active hepatic stellate cells (HSCs) were reported to use aerobic glycolysis as their energy source.^12^ Inhibiting the key enzymes in this pathway, including HK2,^13^ 6‐phosphofructo‐2‐kinase/fructose‐2,6‐bisphosphatase‐3 (PFKFB3)^14^ and pyruvate kinase M2 (PKM2),^15^ could suppress the activation of hepatic stellate cells and the subsequent liver fibrosis, which is the main contributor to liver cirrhosis and HCC.

Sodium butyrate (NaBu) is a sodium salt metabolite of short‐chain fatty acids (SCFAs), which is produced by the gut microbiota NaBu is generally produced by *Firmicutes* and exerts antioxidant, immunomodulatory and anticancer effects.^16^, ^17^ Furthermore, NaBu modulates glycolysis in breast cancer cells,^18^ dendritic epidermal T cells^19^ and lung cancer cells.^20^ According to a previous study, the anti‐HCC effects of NaBu are dependent on the induction of apoptosis; cell cycle arrest and autophagy; and the inhibition of proliferation, migration, invasion and epithelial‐to‐mesenchymal transition.^17^ Activation of the tumour suppressor protein p53,^21^ modulation of β‐catenin,^22^ c‐myc,^23^ mitogen‐activated protein kinase (MAPK), Akt‐mTOR pathway^24^ and inhibition of histone deacetylase^25^ constitute the key mechanisms. Xing et al. reported that in the presence of oxidative stress induced by H_2_O_2_, low‐dose (0.3 mM) NaBu suppresses glycolysis and enhances OXPHOS of the human hepatoblastoma cell line HepG2 to promote cell survival.^26^ However, the effects of NaBu on glycolysis in the HCC cells and whether the anti‐HCC effects of NaBu are related to the modulation of key enzymes in the glycolytic pathway are yet to be elucidated.

In the present study, the results of cell line and animal experiments indicated that NaBu inhibited the proliferation and glycolysis of the HCC cells and induced their apoptosis both *in vivo* and *in vitro*. The modulatory effect of NaBu on glycolysis, apoptosis and proliferation of the HCC cells was related to its inhibition of HK2. NaBu downregulated the expression of HK2 via the c‐myc pathway. Moreover, the compound reduced the sorafenib‐induced upregulation of glycolysis and enhanced the anti‐HCC effect of sorafenib both *in vitro* and *in vivo*. These results are likely to aid us in better understanding the role of butyrate in hepatocellular carcinogenesis and in identifying effective therapeutic strategies for HCC.

## 2 MATERIALS AND METHODS

### 2.1 Reagents

NaBu, sorafenib, dimethyl sulfoxide and puromycin were purchased from Sigma‐Aldrich (St. Louis, MO, USA). The kits used for determining the levels of alanine aminotransferase (ALT), aspartate aminotransferase (AST), lactate, creatinine and glucose were purchased from Jiancheng Bioengineering Institute (Nanjing, China). The Cell Counting Kit (CCK8) was obtained from Epizyme Biotech (Shanghai, China). The Annexin V‐FITC Apoptosis Detection Kit and Annexin V‐PE Apoptosis Detection Kit were purchased from Beyotime (Shanghai, China). Penicillin, streptomycin, nuclear and cytoplasmic protein extraction kits, and reverse transcription and polymerase chain reaction (PCR) kits were obtained from Yeasen (Shanghai, China). High‐glucose Dulbecco's Modified Eagle's Medium (DMEM) and foetal bovine serum (FBS) were bought from HyClone (GE Healthcare, Logan, UT, USA).

### 2.2 Cell culture

The human HCC cell lines, HCC‐LM3, Bel‐7402 and SMMC‐7721; the LO2 normal liver cell line; and the HepG2 human hepatoblastoma cell line^27^ were purchased from the Chinese Academy of Sciences Committee Type Culture Collection cell bank (Shanghai, China). All cell lines were cultured in DMEM supplemented with 10% FBS, 100 U/ml penicillin and 100 g/ml streptomycin at 37°C under a 5% CO_2_ humidified atmosphere.

### 2.3 CCK8 assay

The cells in the logarithmic growth phase were added to the wells of a 96‐well plate at the density of 2 × 10^4^/ml and incubated for 24 h, after which the culture medium was removed and replaced with a fresh medium containing 0, 1, 2, 3, 4 or 5 mmol/L NaBu. The cells were re‐incubated for 24, 48 or 72 h. Next, 10 µl of the CCK8 solution was added to each well, and the cells were incubated for 2 h at 37℃. A microplate reader was used to detect the absorbance at 450 nm. The effects of NaBu on cell viability and the half‐maximum inhibition concentration (IC_50_) of NaBu on different cell lines were calculated using the CalsuSyn software. When exploring the effects of NaBu on the sensitivity of HCC‐LM3 cells to sorafenib *in vitro*, the combination index (CI) and the dose reduction index (DRI) were calculated using the CalsuSyn software.

### 2.4 Colony formation

Cells were seeded at the rate of 800 cells/well in a 6‐well plate and incubated for 5 days. Then, 2, 3 or 4 mmol/L NaBu was added to the cells, and the plates were incubated for another 10 days and then fixed with ethanol and stained with 0.1% crystal violet.

### 2.5 Flow cytometric analysis of apoptosis

Cells at the density of 4 × 10^5^/ml were seeded into 6‐well plates and incubated for 24 h. After which, 3 mmol/L of NaBu was added to the cells and the plate was incubated for another 48 h. Next, the cells were collected and centrifuged at 800×*g* for 5 min. The cells were washed twice with PBS and then treated with 1 × binding buffer containing Annexin‐V/PI for 15 min at room temperature. The cells were assessed by flow cytometer, and the results obtained were analysed with the FlowJo software (FlowJo LLC, Ashland, OR, USA). To investigate the effect of the HK2 overexpression on the apoptosis rate of HCC cells treated with NaBu, the HK2‐overexpressing HCC cells were stained with phycoerythrin (PE) according to the manufacturer's protocol and then detected by flow cytometry. The results obtained were analysed by using the FlowJo software.

### 2.6 Western blotting

Proteins were extracted from the nucleus and cytoplasm using nuclear and cytoplasmic protein extraction kits. The total protein and the nuclear protein were extracted by radioimmunoprecipitation assay lysis in a buffer containing protease inhibitors and phenylmethanesulfonyl fluoride (Epizyme Biotech). The protein concentration was measured using the BCA Kit (Beyotime). Then, 30 μg of the protein was loaded onto sodium dodecyl sulphate‐polyacrylamide gels and transferred onto the polyvinylidene difluoride membranes. The membranes were blocked with 5% bovine serum albumin (BSA) for 1 h at room temperature and incubated with primary antibodies overnight at 4℃ and with secondary antibodies for 1 h at room temperature. Finally, the blots were scanned using an Odyssey two‐colour infrared laser imaging system (LI‐COR Biosciences, Lincoln, NB, USA). The details of the primary antibodies used in the present study are presented in Table S1.

### 2.7 Real‐time (RT) PCR

The primers used in the present study are listed in Table S2. Total RNA was obtained with the TRIzol reagent. cDNA was reverse‐transcribed from total RNA using a reverse transcription kit. Then, RT‐PCR was performed using the 7900 HT Fast PCR System in accordance with the manufacturer's protocol (Yeasen).

### 2.8 Biochemical assays

The lactate levels of the culture medium and the tumour tissues were determined using the Lactate Testing Kit, while the serum levels of ALT, AST and creatinine were detected using appropriate kits, all produced by the Jiancheng Bioengineering Institute (Nanjing, China). The levels of cell glucose consumption were detected as previously described,^28^ and the results were normalized to the protein level.

### 2.9 Plasmid construction, lentivirus packaging, siRNAs construction and infection

The HK2‐overexpressing lentivirus and c‐myc‐overexpressing lentivirus were synthesized by Genechem (Shanghai, China). A full‐length cDNA encoding the HK2 sequence or c‐myc sequence was amplified from 293T cDNA and then cloned into the Ubi‐MCS‐SV40‐EGFP‐IRES‐puromycin vector. Empty vector (EV) was employed as the negative control. In addition, double‐stranded oligonucleotides were cloned into the hU6‐MCS‐Ubiquitin‐EGFP‐IRES‐puromycin vector to generate a recombinant plasmid expressing HK2‐shRNA (sh‐HK2). Nonsense scrambled oligonucleotide (Scramble) was used as the control. HCC‐LM3 and Bel‐7402 cells were seeded into a 12‐well plate at the concentration of 3 × 10^4^/ml. After 24 h, the cells were infected with lentivirus for 12 h. Then, the cells were provided fresh culture medium and then incubated for another 72 h, before observation under a fluorescence microscope. Green fluorescence inside the cell represented a successful transfection. Positive cells were selected after 48 h of puromycin treatment, and the transfection efficiency was analysed by Western blotting.

C‐myc‐specific siRNA‐1 and 2 complementary to c‐myc were synthesized by Genomeditech (Shanghai, China). The target sequences used in this study are shown as follows: c‐myc siRNA‐1, sense 5'‐CCUGAGACAGAUCAGCAACAA (tt)‐3' and antisense 5'‐UUGUUGCUGAUCUGUCUCAGG (tt)‐3'; c‐myc siRNA‐2, sense 5'‐GGAACAAGAAGAUGAGGAA (tt)‐3' and antisense 5'‐UUCCUCAUCUUCUUGUUCC (tt)‐3'. The siRNAs were transfected into HCC cells using Lipofectamine 2000 (Invitrogen, USA) in accordance with the manufacturer's protocol. The transfection efficiency was verified by Western blotting.

### 2.10 Animal experiments

Four‐week‐old male BALB/C nude mice were purchased from Shanghai SLAC Laboratory Animal Center (Shanghai, China). The mice had free access to clean water and food and were housed under standard animal laboratory conditions. All animal experiments were conducted as per the National Institutes of Health Guidelines for the Care and Use of Laboratory Animals. The current study was approved by the Animal Care and Use Committee of Shanghai Tongji University (Shanghai, China). Logarithmic growth phase LM3 cell suspensions (5 × 10^6^) in a serum‐free DMEM were injected into the upper flank region of each mouse. After 4 days, when the tumours became measurable, the mice were randomly assigned into two groups (*n* = 4 per group): (1) normal control (NC): mice that received saline via gavage once daily for 16 days, and (2) NaBu: mice that received 200 mg/kg NaBu via gavage once daily for 16 days. The weight of the mice and the tumour volume were recorded every 4 days. Tumour volume was calculated as follows: (mm^3^) = 0.5 × (major axis) × (minor axis)^2^.

For the combination treatment with sorafenib, 12 mice were randomly assigned to four groups (*n* = 3): (1) the NC group mice received saline via gavage once daily for 16 days; (2) the NaBu group mice received 200 mg/kg NaBu via gavage once daily for 16 days; (3) the Sora group mice received 10 mg/kg sorafenib via gavage once daily for 16 days; and (4) the Sora + NaBu group mice received both 10 mg/kg sorafenib and 200 mg/kg NaBu via gavage once daily for 16 days.

After 20 days, the mice were anaesthetized with 1.25% pentobarbital and sacrificed. Their tumours were removed and photographed. Their blood, liver, kidney, lung and heart were collected for toxicity analyses.

### 2.11 Haematoxylin and eosin staining

The tumour tissues were fixed with paraformaldehyde and then embedded in paraffin. The paraffin blocks were cut into 5‐μm‐thick sections and subjected to haematoxylin and eosin staining.

### 2.12 Immunohistochemical (IHC) staining

Tumour sections (5‐μm thick) were dewaxed and dehydrated. After antigen retrieval and blocking, the sections were incubated with primary antibodies against Ki67, HK2, PFK1, LDH‐A and c‐myc. These sections were then incubated with appropriate secondary antibodies. The positively stained areas were observed by light microscopy.

### 2.13 TUNEL assay

Tumour sections (5‐μm thick) were dewaxed, dehydrated and digested with proteinase K. Then, the sections were incubated with the TUNEL reaction mixture. TUNEL‐positive cells were observed and imaged by light microscopy.

### 2.14 Statistical analysis

All experiments were performed at least thrice. Data are shown as mean ± standard deviation (SD). Differences between the groups were analysed by Student's *t*‐tests or one‐way analysis of variance. *p* < 0.05 was considered to indicate statistical significance.

## 3 RESULTS

### 3.1 NaBu inhibits cell proliferation and induces apoptosis both *in vitro* and *in vivo*

**FIGURE 1:**
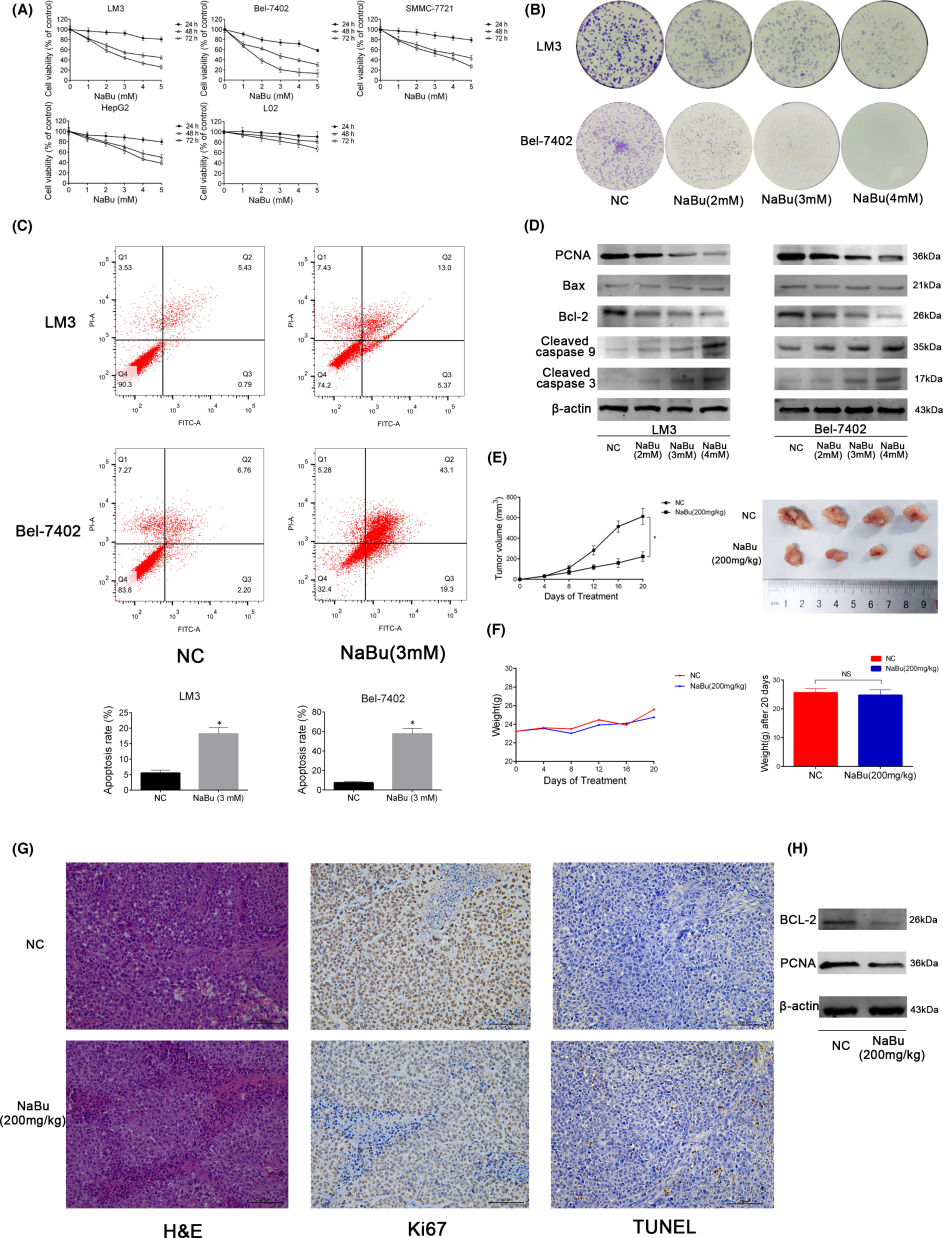
Effects of NaBu on proliferation and apoptosis of HCC *in vitro* and *in vivo*. (A) The CCK8 assay was performed to detect the viability of cells treated with NaBu (0–5 mM) for 24–72 h. (B) Colony formation by HCC‐LM3 and Bel‐7402 cells. (C) The apoptosis rate of HCC‐LM3 and Bel‐7402 cells after NaBu treatment for 48 h, determined by flow cytometry (*n* = 3, *p < 0.05 for NC vs. NaBu [3 mM]). (D) The protein expression of PCNA, Bax, Bcl‐2, cleaved caspase 3 and cleaved caspase 9, detected by Western blotting. (E) Tumour volume was recorded at the indicated time points (*n* = 4, *p < 0.05 for NC vs. NaBu). The gross manifestation of tumours. (F) Changes in mouse body weight were recorded at the indicated time points (*n* = 4, *p* > 0.05). (G) haematoxylin and eosin, TUNEL and Ki67 staining of tumour sections in the NC and NaBu groups (magnification 200×). (H) The protein expression of Bcl‐2 and PCNA in the mouse tumour tissues detected by Western blotting

The toxicity of NaBu on the HCC and normal liver cell lines was determined. The CCK8 assay was performed to detect the effects of NaBu on the proliferation of the HCC cell lines HCC‐LM3, Bel‐7402 and SMMC‐7721; the hepatoblastoma cell line HepG2; and the normal liver cell line LO2. NaBu inhibited the proliferation of all three HCC cell lines and the HepG2 cell line in a time‐ and dose‐dependent manner (Figure 1A). Moreover, the inhibitory effect of NaBu on the LO2 cells line was inferior to that on the HCC cell lines and the HepG2 cell line. The IC_50_ values of NaBu on HCC‐LM3, Bel‐7402, SMMC‐7721, HepG2 and LO2 cell lines at 48 h were 3.91, 2.61, 4.18, 5.25 and 19.68 mM, respectively, which showed that the HCC‐LM3 and Bel‐7402 cell lines were more sensitive to NaBu treatment than the other cell lines used in the present study. The results of the colony formation assay indicated that NaBu treatment hampered the proliferation and colony‐forming ability of the HCC‐LM3 and Bel‐7402 cell lines (Figure 1B). Moreover, the results from Western blotting demonstrated that NaBu suppressed the protein expression of the proliferating cell nuclear antigen (PCNA) in the HCC‐LM3 and Bel‐7402 cell lines (Figure 1D), which further confirmed that NaBu inhibited the proliferation of the HCC cells *in vitro*.

Flow cytometric analysis indicated that 3 mM NaBu increased the apoptosis rate in the HCC‐LM3 and Bel‐7402 cell lines (Figure 1C). Bcl‐2 is an anti‐apoptotic protein whereas Bax, cleaved caspase 3 and cleaved caspase 9 are pro‐apoptotic proteins. Western blotting analysis revealed that NaBu decreased the expression of Bcl‐2 but increased the expression of cleaved caspase 9 and 3 (Figure 1D). NaBu did not affect the expression of Bax (Figure 1D). These results suggested that NaBu induced the apoptosis of the HCC cells *in vitro*. Furthermore, the results of Western blotting showed that NaBu did not affect the expression of Bcl‐2, Bax or cleaved caspase 3 in the LO2 cell line (Figure S1), which indicated that NaBu had a lower capacity to induce apoptosis in the LO2 cell line.

Subsequently, a xenograft model was generated by injecting the HCC‐LM3 cell line to investigate the effects of NaBu on proliferation, apoptosis and glycolysis of the HCC cells *in vivo*. The gavage of 200 mg/kg NaBu significantly decreased the tumour volume (Figure 1E), whereas it did not affect the weight of the mice (Figure 1F). Haematoxylin and eosin staining showed that there were more necrotic lesions in the NaBu group than in the NC group. TUNEL assay exposed that there were more apoptotic cells in the NaBu group than in the NC group. Ki67 has been viewed as a proliferation marker in cancer cells. In the present study, Ki67 staning showed that there were more positive cells in the NC group than in the NaBu group (Figure 1G). The results of Western blotting showed that 200 mg/kg NaBu inhibited the expressions of Bcl‐2 and PCNA in the mouse tumour tissues (Figure 1H). These results indicated that NaBu suppressed the proliferation and induced the apoptosis of the HCC cells *in vivo*.

Moreover, NaBu did not affect the mouse serum levels of ALT, AST or creatinine (Figure S2A). Furthermore, no obvious pathological changes were observed in the liver, lung, heart or kidney sections of the NaBu‐treated mice (Figure S2B), which implied that the gavage of 200 mg/kg of NaBu daily for 16 days did not harm these organs.

Collectively, the above results show that NaBu induces apoptosis and suppresses the proliferation of the HCC cells both *in vivo* and *in vitro*.

### 3.2 NaBu inhibits aerobic glycolysis in the HCC cells both *in vitro* and *in vivo*

**FIGURE 2:**
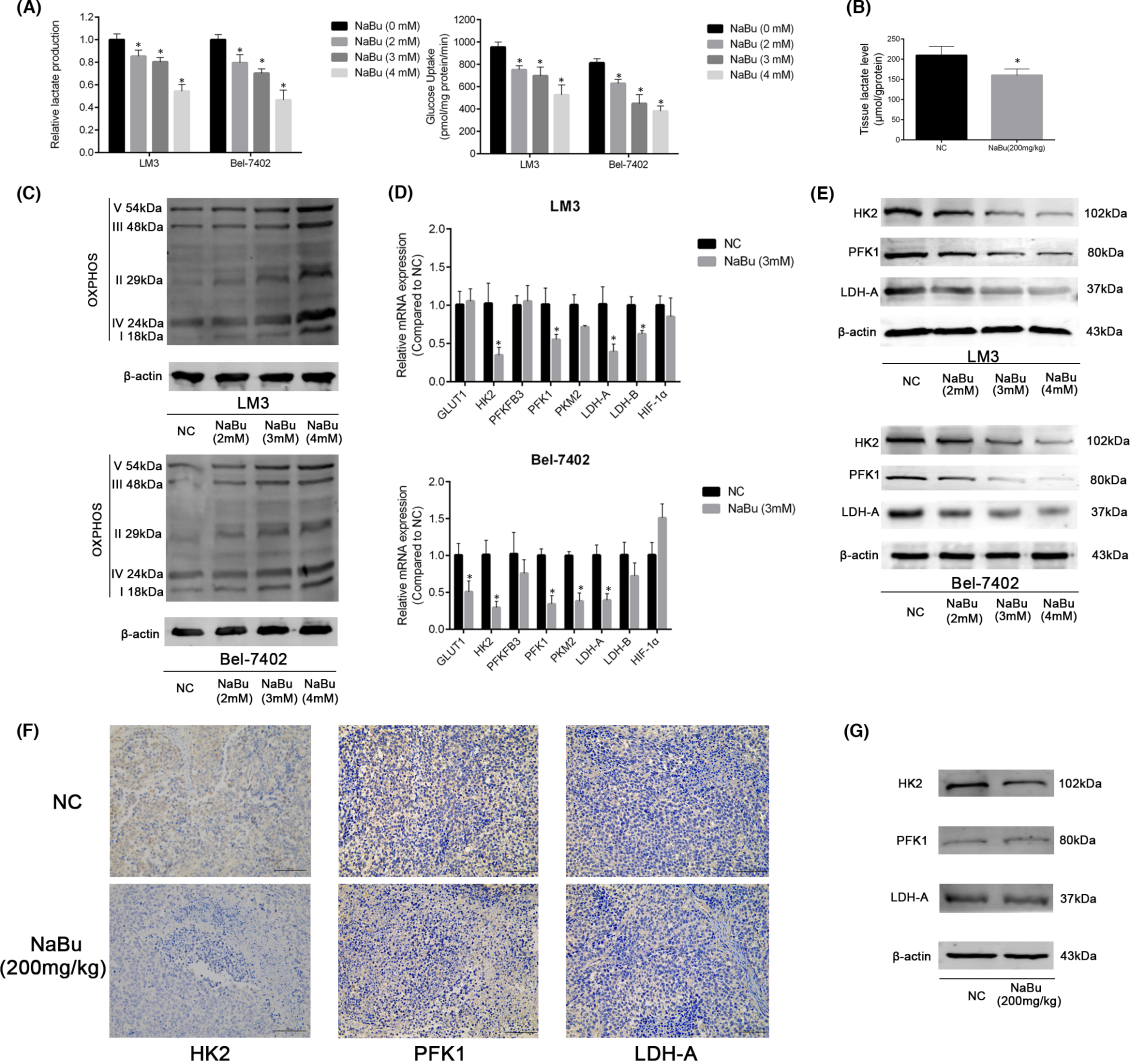
Effects of NaBu on glycolysis of HCC cells *in vitro* and *in vivo*. (A) The effects of NaBu on lactate production and glucose consumption in HCC‐LM3 and Bel‐7402 cells (*n* = 3, *p < 0.05 vs. the NaBu [0 mM] group). (B) The effects of NaBu on the lactate level of mouse tumour tissues (*n* = 4, *p < 0.05 vs. the NC group). (C)Western blotting analysis of OXPHOS in HCC‐LM3 and Bel‐7402 cells. (D) qPCR analysis of 8 aerobic glycolysis‐related genes (*n* = 3, *p < 0.05 vs. the NC group). (E)Western blotting analysis of HK2, PFK1 and LDH‐A in HCC‐LM3 and Bel‐7402 cells. (F) IHC staining of tumour tissue for HK2, PFK1 and LDH‐A (magnification 200×). (G) The protein expressions of HK2, PFK1 and LDH‐A in the mouse tumour tissues were detected by Western blotting

Next, the effects of NaBu treatment on aerobic glycolysis in the NaBu‐sensitive cell lines HCC‐LM3 and Bel‐7402 were determined. The results showed that NaBu treatment for 48 h inhibited lactate production and glucose consumption by the HCC‐LM3 and Bel‐7402 cell lines in a dose‐dependent manner (Figure 2A). Moreover, 200 mg/kg NaBu decreased the lactate level in the mouse tumour tissues (Figure 2B). However, NaBu enhanced the expression of mitochondrial OXPHOS in a dose‐dependent manner in both the HCC cell lines (Figure 2C). The mRNA levels of eight glycolysis‐related genes were determined using RT‐PCR in the HCC‐LM3 and Bel‐7402 cell lines. The results signified that the mRNA expressions of HK2, phosphofructokinase‐1 (PFK1) and LDH‐A were all significantly suppressed in both HCC‐LM3 and Bel‐7402 cell lines upon treatment with 3 mM NaBu for 48 h (Figure 2D). Among these three genes, the mRNA level of HK2 decreased the most in both cell lines after NaBu treatment. HK2, PFK1 and PKM2 have been identified to be the three rate‐limiting enzymes of aerobic glycolysis in the HCC cells.^7^ Lactate dehydrogenase A (LDH‐A) converts pyruvate to lactate, and its activity is positively correlated with aerobic glycolysis.^29^ The results of Western blotting showed that NaBu treatment for 48 h suppressed the expressions of HK2, PFK1 and LDH‐A in the HCC‐LM3 and Bel‐6402 cell lines in a dose‐dependent manner (Figure 2E). The results of IHC staining and Western blotting indicated that 200 mg/kg NaBu inhibited the expression of HK2 in the mouse tumour tissues. However, 200 mg/kg NaBu did not have any effect on the expressions of PFK1 and LDH‐A in the mouse tumour tissues (Figure 2F and G). The above results demonstrated that NaBu treatment suppresses aerobic glycolysis in the HCC cells both *in vivo* and *in vitro*.

### 3.3 The modulation of aerobic glycolysis, proliferation, and apoptosis of the HCC cells by NaBu is associated with the inhibition of HK2

In the present study, among the eight glycolysis‐related genes, the mRNA level of HK2 witnessed the highest decrease in the HCC‐LM3 and Bel‐7402 cell lines after NaBu treatment. This treatment suppressed the protein expression of HK2 in the HCC cells both *in vitro* and *in vivo*.

**FIGURE 3:**
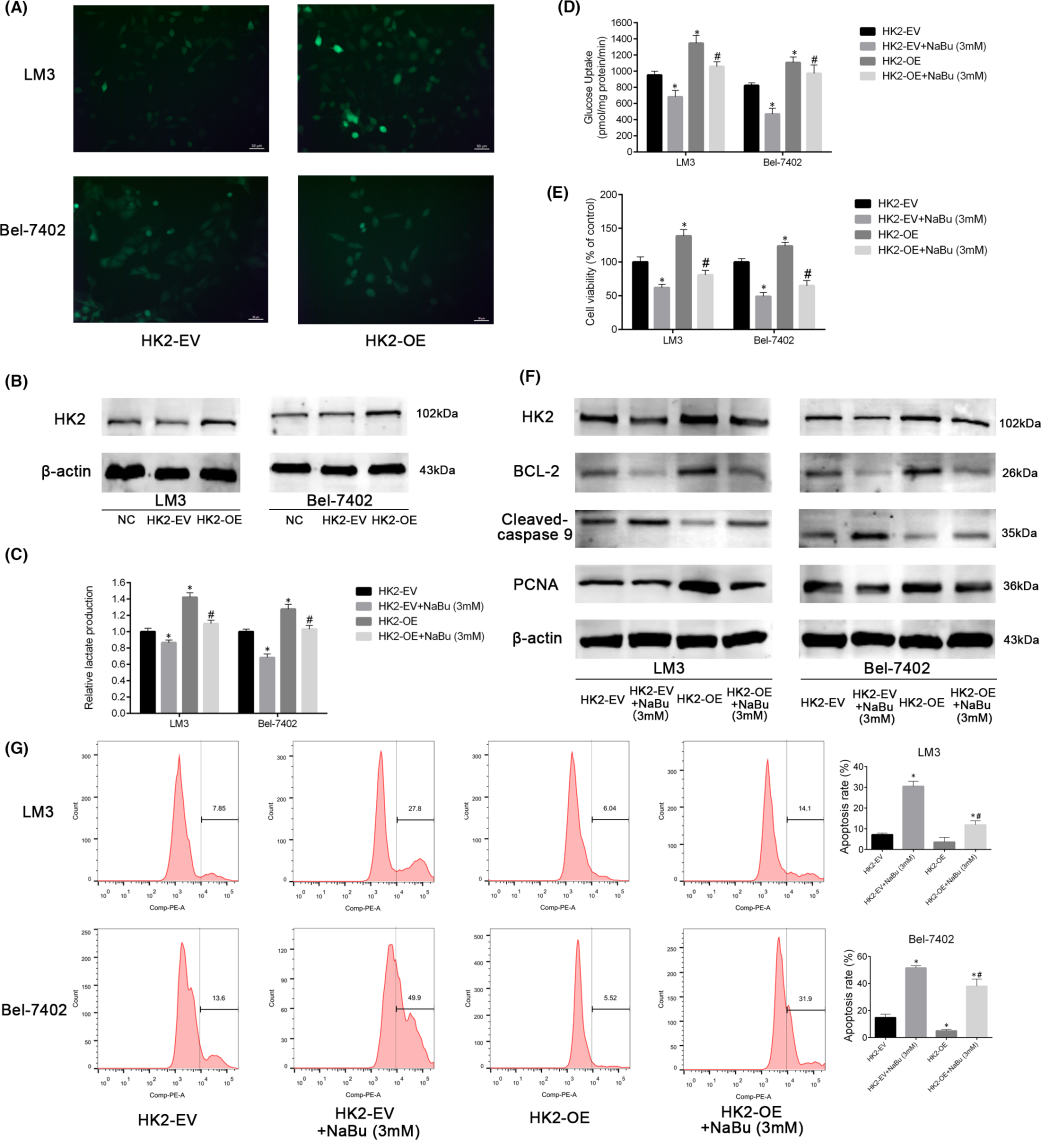
NaBu regulated glycolysis, proliferation and apoptosis of HCC cells by inhibiting HK2 expression. (A) The fluorescence of HCC cells after transfection with empty vector or HK2‐OE lentiviruses (magnification, 200×). (B) The transfection efficiency was verified by Western blotting. (C and D) Effects of 3 mM NaBu on the lactate production and glucose consumption of HK2‐EV or HK2‐OE HCC cells (*n* = 3, *p < 0.05 vs. the HK2‐EV group, #*p* < 0.05 vs. the HK2‐EV + NaBu (3 mM) group). (E) Effects of 3 mM NaBu treatment for 48 h on the cell viability of HK2‐EV or HK2‐OE HCC cells (*n* = 3, *p < 0.05 vs. the HK2‐EV group, #*p* < 0.05 vs. HK2‐EV + NaBu group). (F) Effects of 3 mM NaBu on the expression of HK2, Bcl‐2, cleaved caspase 9, and PCNA in HK2‐EV or HK2‐OE HCC cells. (G) Effects of 3 mM NaBu on the apoptosis rate of HK2‐EV or HK2‐OE HCC cells (*n* = 3, *p < 0.05 vs. the HK2‐EV group, #*p* < 0.05 vs. HK2‐EV + NaBu (3 mM) group)

Hence, whether the induction of apoptosis and the inhibition of aerobic glycolysis and proliferation of the HCC cells by NaBu were related to the downregulation of HK2 was investigated. For this study, the HCC‐LM3 and Bel‐7402 cell lines were transfected with lentivirus overexpressing (OE) either EV or HK2. The efficiency of transfection was verified using green fluorescent protein imaging and Western blotting (Figure 3A and B). The results of Western blotting indicated that the protein expression of HK2 in the HK2‐EV and HK2‐OE HCC cell lines could be downregulated by NaBu (Figure 3F). As shown in Figure 3D and E, compared with the HK2‐EV group, the lactate production and glucose consumption in both the HCC cell lines were enhanced by HK2 expression. Moreover, the inhibitory effects of NaBu on lactate production and glucose consumption were impaired in the HK2‐OE group compared with the HK2‐EV group (Figure 3C and D). These results suggest that HK2 is essential for the inhibition of aerobic glycolysis upon NaBu treatment in the HCC cells.

As shown in Figure 3E, the viability of the HCC cells in the HK2‐OE group was higher than that in the HK2‐EV group. Furthermore, HK2 overexpression reversed the decrease in proliferation induced by treatment with 3 mM NaBu for 48 h. The effect of HK2 overexpression on PCNA expression agreed with the results of the CCK8 assay (Figure 3F). Meanwhile, Western blotting demonstrated that HK2 overexpression enhanced the expression of Bcl‐2 and inhibited the expression of cleaved caspase 9 in the HCC cells, which indicated the downregulation of apoptosis. Furthermore, HK2 overexpression attenuated NaBu‐induced apoptosis in both the HCC cell lines (Figure 3F). The results of flow cytometry alluded that HK2 overexpression lowered the rate of apoptosis in the LM3 and Bel‐7402 cell lines treated with 3 mM NaBu (Figure 3G).

These findings imply that HK2 is essential for the inhibition of aerobic glycolysis and cell proliferation and for the induction of apoptosis upon NaBu treatment in the HCC cells.

### 3.4 NaBu inhibits HK2 via the c‐myc pathway

**FIGURE 4:**
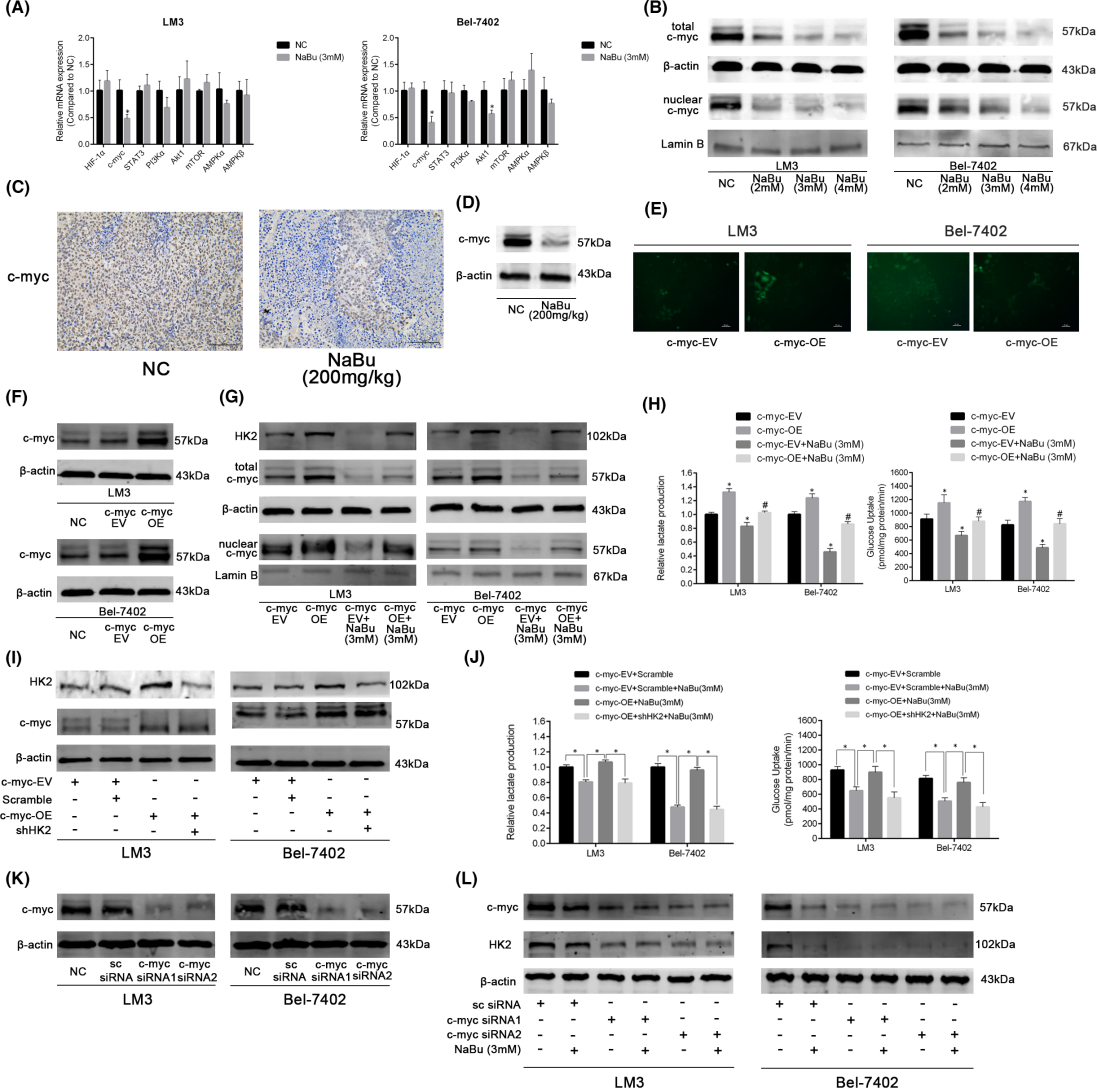
NaBu downregulated HK2 expression via the c‐myc pathway. (A) qRT–PCR analysis of the effect of 3 mM NaBu treatment for 48 h on the expression of genes associated with HK2 modulation in HCC‐LM3 and Bel‐7402 cells (*n* = 3, *p < 0.05 vs. the NC group). (B) Effect of 48 h 3 mM NaBu treatment on the total and nuclear expression of c‐myc in HCC cells. (C) IHC staining of c‐myc in mouse tumour tissues (magnification 200×). (D)The expression of c‐myc in the mouse tumour tissues detected by Western blotting. (E) The fluorescence of HCC cells after transfection with empty vector or c‐myc‐OE lentiviruses (magnification, 200×). (F) The transfection efficiency was verified by Western blotting. (G) Effects of 3 mM NaBu on the expression of HK2, total c‐myc, and nuclear c‐myc in c‐myc‐EV or c‐myc‐OE HCC cells. (H) Effects of 3 mM NaBu on the lactate production and glucose consumption of c‐myc‐EV or c‐myc‐OE HCC cells (*n* = 3, *p < 0.05 vs. the c‐myc‐EV group, #*p* < 0.05 vs. the c‐myc‐EV + NaBu (3 mM) group). (I) The transfection efficiency was verified by Western blotting. (J) The effects of HK2 knockdown on the lactate production and glucose consumption of c‐myc‐OE HCC cells treated with 3 mM NaBu (*n* = 3, *p < 0.05). (K) The transfection efficiency was verified by Western blotting. (L) Effects of 3 mM NaBu on the expression of c‐myc and HK2 in c‐myc siRNA knockdown HCC cells

Next, the signalling pathways involved in NaBu‐induced HK2 inhibition were explored. The HK2 expression was modulated by several signalling pathways and transcription factors, including c‐myc, HIF‐1α, PI3K/Akt, AMPK and STAT3.^7^ RT‐PCR was performed to investigate the effects of NaBu treatment on the mRNA levels of these genes. The findings revealed that the mRNA expression of c‐myc was significantly inhibited in both HCC‐LM3 and Bel‐7402 cell lines upon treatment with 3 mM NaBu for 48 h (Figure 4A). Therefore, whether the inhibition of HK2 by NaBu was dependent on its modulation of the c‐myc pathway was investigated.

As shown in Figure 4B, the protein expressions of total c‐myc and nuclear c‐myc in the HCC‐LM3 and Bel‐7402 cell lines were inhibited by NaBu treatment for 48 h in a dose‐dependent manner. The outcomes of IHC staining and Western blotting suggested that 200 mg/kg NaBu inhibited the protein expression of c‐myc in the mouse tumour tissues (Figure 4C and D). These results showed that NaBu inhibited the c‐myc pathway both *in vitro* and *in vivo*.

Subsquently, a c‐myc‐overexpressing lentivirus was utilized to verify whether the inhibition of HK2 was associated with the downregulation of the c‐myc pathway. The efficiency of transfection was validated using green fluorescent protein imaging and Western blotting (Figure 4E and F). After infection with the c‐myc‐overexpressing lentivirus, the expressions of HK2, total c‐myc and nuclear c‐myc were enhanced in both the HCC cell lines. Moreover, the reduction in the expressions of HK2, total c‐myc and nuclear c‐myc by NaBu was reversed by c‐myc overexpression (Figure 4G). As depicted in Figure 4H, c‐myc overexpression enhancedlactate production and glucose uptake in the HCC‐LM3 and Bel‐7402 cell lines, and attenuated the inhibitory effects of NaBu on lactate production and glucose uptake in both the cell lines. Moreover, the effects of c‐myc overexpression on aerobic glycolysis of the HCC cells treated with NaBu were reversed by the knockdown of HK2 (Figure 4I and J). To further understand how HK2 inhibition was regulated by the c‐myc pathway, the expression of c‐myc was ablated in the HCC cells via siRNA transfection (Figure 4k). The results of Western blotting showed that HK2 protein expression was not affected by NaBu in the c‐myc siRNA knockdown HCC cells, which demonstrated that c‐myc was the target of NaBu when inhibiting HK2 in the HCC cells (Figure 4L). The above results imply that NaBu downregulates HK2 expression via the c‐myc pathway, thereby suppressing aerobic glycolysis in the HCC cells.

### 3.5 NaBu enhances the chemosensitivity of sorafenib in the HCC cells both *in vitro* and *in vivo*

Sorafenib is a first‐generation targeted therapeutic drug for advanced HCC. Glucose consumption and lactate export are increased and mitochondrial OXPHOS is impaired in the HCC cells after sorafenib treatment.^30^ Moreover, lactate can function as a direct signal instructing cancer‐associated fibroblast that secretes hepatocyte growth factor, thus reducing the sensitivity of the tumours to sorafenib treatment.^31^ Inhibition of key enzymes in the glycolytic pathway via lentivirus transfection or drugs enhances the sensitivity of the HCC cells to sorafenib.^10^, ^32^, ^33^ In the present study, NaBu was found to inhibit the glycolysis influx of the HCC cells. However, the effect of NaBu treatment on the sensitivity of the HCC cells to sorafenib remains to be investigated.

**FIGURE 5:**
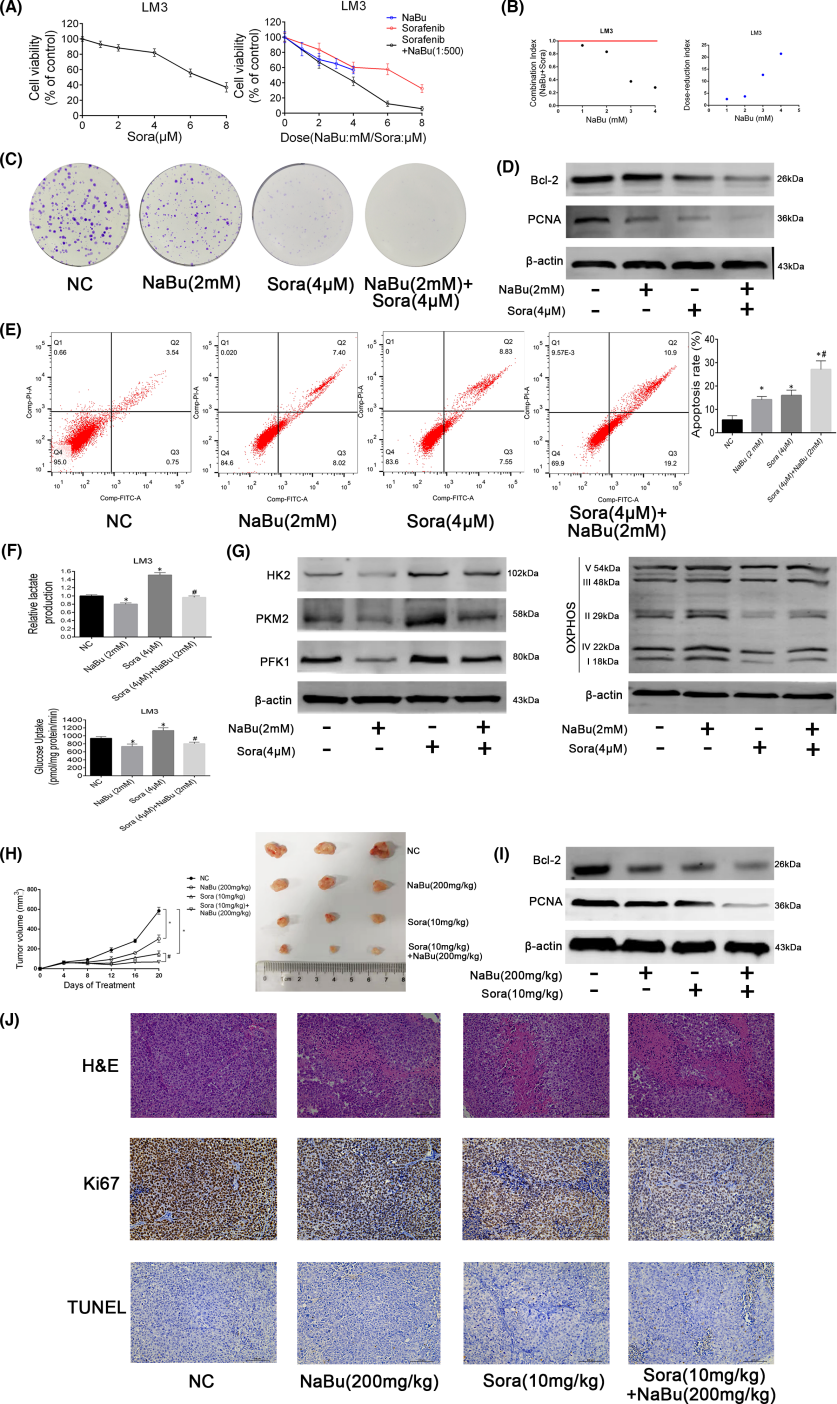
NaBu enhanced the chemosensitivity of sorafenib in HCC cells *in vivo* and *in vitro*. (A) HCC‐LM3 cells were treated with sorafenib or/and NaBu for 48 h. Cell viability was assessed by CCK8 assay (*n* = 3, data are shown as mean ± SD). (B) CI and DRI were calculated using CalsuSyn software. (C) Effects of NaBu or/and sorafenib on the colony formation of HCC‐LM3 cells. (D) Effects of 48 h NaBu or/and sorafenib treatment on the expression of Bcl2 and PCNA in HCC‐LM3 cells. (E) Effects of 48 h NaBu or/and sorafenib treatment on the apoptosis rate of HCC‐LM3 cells (*n* = 3, *p < 0.05 vs. the NC group, #*p* < 0.05 vs. the Sora (4 μM) group). (F) Effects of 48 h NaBu or/and sorafenib treatment on the lactate production and glucose uptake of HCC‐LM3 cells (*n* = 3, *p < 0.05 vs. the NC group, #*p* < 0.05 vs. the Sora (4 μM) group). (G) Effects of 48 h NaBu or/and sorafenib treatment on the protein expression of HK2, PKM2, PFK1 and OXPHOS in HCC‐LM3 cells. (H) Tumour volume was recorded at the indicated time points (*n* = 3, *p < 0.05 vs. the NC group, #*p* < 0.05 vs. the Sora group). (I) The expression of Bcl‐2 and PCNA detected by Western blotting. (J) Haematoxylin and eosin, Ki67 and TUNEL staining of tumour sections (magnification 200×)

First, the HCC‐LM3 cell line was treated with different concentrations of sorafenib for 48 h. The IC_50_ of sorafenib on the cell line at 48 h was 7.36 μM (Figure 5A). The IC_50_ of NaBu on the cell line at 48 h was 3.91 mM (Figure 1A). Thus, the ratio of the IC_50_ of sorafenib to that of NaBu on the cell line was approximately 1:500. Next, different concentrations of sorafenib were combined with NaBu (at a fixed ratio of 1:500) to determine whether NaBu can enhance the sensitivity of the HCC‐LM3 cell line to sorafenib. As shown in Figure 5A, compared with sorafenib or NaBu alone, the combination of sorafenib and NaBu (1:500) exerted a greater cytotoxic effect on the HCC‐LM3 cell line. Moreover, the results of the median dose‐effect analysis indicated that the combination index (CI) in the HCC‐LM3 cells was <1, which implied the synergistic effect of sorafenib and NaBu (Figure 5B). The DRI of NaBu was >1, which suggested that NaBu can lower the dose of sorafenib (Figure 5B). Moreover, the inhibitory effects of sorafenib on the colony‐forming ability and PCNA expression of the HCC‐LM3 cell line were further enhanced by NaBu treatment (Figure 5C and D). These results denote that NaBu enhances the inhibitory effects of sorafenib on the proliferation of the HCC cells.

Flow cytometric analysis showed that 2 mM NaBu and 4 μM sorafenib exerted comparable effects on the apoptosis rate of the HCC‐LM3 cell line, and NaBu treatment was found to enhance the apoptosis induced by sorafenib (Figure 5E). Moreover, the reduction in Bcl2 expression following sorafenib treatment in the HCC‐LM3 cell line was further suppressed by NaBu (Figure 5D).

The lactate production, glucose uptake and protein expression of the rate‐limiting enzymes involved in the glycolytic pathway were improved in the HCC‐LM3 cell line after sorafenib treatment, whereas mitochondrial OXPHOS was impaired (Figure 5F and G). These results were consistent with those obtained from our previous study.^28^ However, these trends were reversed upon NaBu treatment (Figure 5F and G). The above findings signify that NaBu impairs the enhancement of aerobic glycolysis induced by sorafenib in the HCC cells and increases the sensitivity of HCC cells to sorafenib *in vitro*.

The results of *in vivo* studies showed that mice treated with NaBu or sorafenib treatment alone had smaller tumour volumes than untreated mice after 16 days of therapy. The combined administration of NaBu and sorafenib strengthened this effect (Figure 5H). Haematoxylin and eosin staining revealed that the combination treatment of NaBu and sorafenib increased the necrosis of the HCC cells in cpmparison with NaBu or sorafenib alone (Figure 5J). The results of Western blotting, TUNEL staining and Ki67 staining demonstrated that the inhibition of proliferation and the induction of apoptosis in the HCC cells by sorafenib were further enhanced by NaBu *in vivo* (Figure 5I and J).

Collectively, these results establish that NaBu treatment enhances the chemosensitivity of the HCC cells to sorafenib both *in vitro* and *in vivo*.

## 4 DISCUSSION

In the 1920s, Warburg et al. observed that rat liver carcinoma tissues consumed higher amounts of glucose than the surrounding normal liver tissues. Moreover, the liver carcinoma tissues tended to catabolize glucose to lactate via glycolysis instead of converting it to CO_2_ and H_2_O via the citric acid cycle even in the presence of oxygen.^3^ This phenomenon termed the ‘Warburg effect’ or aerobic glycolysis has been observed in HCC,^4^ lung cancer^34^ and other kinds of cancer. In aerobic glycolysis, 2 moles of ATP are produced from 1 mole of glucose. In contrast, 36 moles of ATP are produced from 1 mole of glucose in mitochondrial OXPHOS.^35^ Although aerobic glycolysis seems inefficient in terms of ATP production, the speed of ATP production via aerobic glycolysis is much higher than that via mitochondrial OXPHOS per unit time.^7^ High‐speed glucose fermentation via aerobic glycolysis helps cancer cells consume more glucose than normal cells.^36^ This rapid ATP generation via aerobic glycolysis aids in the proliferation of the cancer cells.^37^ Glycolytic intermediates, such as dihydroxyacetone phosphate and 3‐phosphoglycerate, support the biosynthetic programs of the cancer cells and promote their proliferation and growth.^38^ Moreover, lactate production via aerobic glycolysis acidifies the tumour microenvironment, which enhances immune evasion, invasion, metastasis, angiogenesis, drug resistance and radioresistance of the cancers.^7^, ^38^, ^39^ A recent review has systematically summarized the noncanonical functions of key enzymes involved in the glycolytic pathway.^8^ For instance, HK2 regulates autophagy and mitochondria‐dependent apoptosis. PKM2 regulates mitosis, cell cytokinesis, homologous recombination repair, apoptosis, tumour cell exosomes and ectosomes, and PFKFB3 regulates DNA repair.^8^ Hence, targeting the enzymes that play a key role in aerobic glycolysis could regulate various phenotypes of the cancer cells and has been regarded as a novel HCC treatment strategy.^40^ Preclinical studies have signified that glycolytic inhibitors, such as chrysin (targeting HK2),^41^ 2‐DG (targeting HK2),^42^, ^43^ proanthocyanidin B2 (targeting PKM2),^28^ methyl jasmonate (targeting HK2)^44^ and genistein (targeting HIF‐1α),^45^ suppress proliferation and induce apoptosis of the HCC cells. Moreover, these glycolytic inhibitors augment the anti‐HCC effects of sorafenib. Xing et al.^46^ reported that emodin inhibits the expressions of HK2, PKM2 and LDH‐A, thus suppressing the proliferation of HepG2 cells.

SCFAs, which have fewer than seven carbon atoms, are produced via the fermentation of dietary fibres by the intestinal flora.^47^ SCFAs are the main energy source of the intestinal epithelial cells, and excess SCFAs are transported to the liver via the portal vein.^48^ NaBu is a sodium salt of the SCFA butyrate. Experimental studies have shown that NaBu exerts a protective effect against various liver diseases, including autoimmune hepatitis,^49^ liver fibrosis^50^ and non‐alcoholic fatty liver disease.^51^ NaBu exerts dual effects on the HCC cells, that is a low‐dose promotes their proliferation,^52^ whereas a high‐dose inhibits their proliferation, invasion and metastasis. Moreover, a high dose induces G0/G1 arrest, apoptosis and autophagy of the HCC cells, thus exerting anti‐HCC effects.^17^, ^23^, ^24^, ^52^ NaBu has been reported to inhibit aerobic glycolysis in the lung^20^ and breast cancer cells.^18^ However, the effects of NaBu on aerobic glycolysis in the HCC cells and whether these effects are related to the modulation of key enzymes in the glycolytic pathway are yet to be unravelled. In the present study, NaBu was found to apoptosis and inhibit the proliferation of the NaBu‐sensitive HCC cell lines HCC‐LM3 and Bel‐7402 in a dose‐dependent manner *in vitro* (Figure 1). However, NaBu had only a low ability to induce apoptosis of the LO2 cell line *in vitro* (Figure S1). The results of the animal experiments alluded that 200 mg/kg NaBu induced apoptosis and inhibited the proliferation of the HCC‐LM3 cell line (Figure 1). However, it did not have any effects on mouse body weight, liver function, kidney function or pathological manifestations in the major organs (Figure S2). NaBu inhibited lactate production and glucose uptake by the HCC‐LM3 and Bel‐7402 cell lines. The protein expressions of HK2, PFK1 and LDH‐A in the HCC‐LM3 and Bel‐7402 cell lines were inhibited by NaBu treatment, which established the inhibitory effects of NaBu on aerobic glycolysis in the HCC cells *in vitro*. Moreover, NaBu enhanced mitochondrial OXPHOS in the HCC cells *in vitro*. A concentration of 200 mg/kg NaBu lowered the lactate levels and the expression of HK2 in the mouse tumour tissues (Figure 2). These results assert that NaBu inhibits aerobic glycolysis in the HCC cells both *in vitro* and *in vivo*.

HK2 is the first rate‐limiting enzyme in the glycolytic pathway and catalysed the conversion of glucose to glucose‐6‐phosphate.^53^ Five isoforms of HK(HK1, HK2, HK3, HK4 and HKDC1) have been identified in humans.^54^ In normal hepatocytes, HK4 or glucokinase is the major isoform.^55^ However, in the HCC cells, HK2 is usually the only expressed HK. This isoform is not expressed in most normal adult tissues, including hepatocytes.^56^ Moreover, no adverse physiological effects have been found in mice with systematic HK2 deletion.^57^ DeWaal et al. reported that the upregulation of HK2 begins during liver cirrhosis, increases in dysplasia and reaches peaks in HCC.^56^ Furthermore, the upregulation of HK2 and the enhancement of glycolysis have been reported to play pertinent roles in the activation of HSCs and liver fibrosis,^13^, ^58^ which is the main contributor to liver cirrhosis and HCC.^59^ These findings show that targeting HK2 could be an excellent and safe strategy for treating HCC, even in the early phase of hepatocellular carcinogenesis. HK2 binds to VDAC1 on the outer mitochondrial membrane via its hydrophobic N terminal, which augments ATP production and promotes the proliferation of the HCC cells.^60^, ^61^ Moreover, the binding of HK2 and VDAC1 suppresses the binding of Bax to VDAC1 and the release of cytochrome C, which prevents the HCC cells from mitochondria‐associated apoptosis.^41^ Hepatic HK2 deletion has been reported to suppress diethylnitrosamine‐induced hepatocarcinogenesis in mice. Knockdown of HK2 in the HCC cells inhibited the proliferation and aerobic glycolysis while enhancing mitochondrial OXPHOS and apoptosis. Moreover, inhibition of HK2 increased the sensitivity of the HCC cells to sorafenib and metformin.^56^ 2‐Deoxy‐d‐glucose (2‐DG), a synthetic glucose analog that can inhibit HK2 activity, has been shown to inhibit the proliferation, metastasis and invasion of the HCC cells and induce their apoptosis.^42^, ^43^ Moreover, 2‐DG enhances the sensitivity of the HCC cells to sorafenib.^42^ Xu et al.^41^ discovered that chrysin suppresses glycolysis in the HCC cells by inhibiting HK2. Moreover, chrysin reduces HK2 combined with mitochondrial VDAC1, thereby inducing apoptosis. Chrysin inhibits HCC‐LM3 xenograft growth and HK2 expression *in vivo*. Li et al.^44^ reported that methyl jasmonate (MJ) decreases the mRNA and protein expression of HK2 but does not affect HK2 activity. MJ suppresses the growth of the HCC cells by inhibiting glycolysis. Moreover, MJ detaches HK2 from VDAC1, which leads to the loss of mitochondrial function and in turn induces apoptosis and necrosis of the HCC cells. MJ enhances the anti‐HCC effects of sorafenib both *in vitro* and *in vivo*. The abovementioned findings strongly indicate the potential of HK2 as a drug target for HCC.

In the present study, among the eight glycolysis‐related genes, the mRNA expression of HK2 was reduced to the greatest extent in both HCC‐LM3 and Bel‐7402 cell lines after NaBu treatment. The protein expression of HK2 in the HCC cells was inhibited by NaBu treatment both *in vitro* and *in vivo*. HK2 overexpression by lentivirus transfection impaired the inhibitory effects of NaBu on lactate production and glucose uptake in both the HCC cell lines. How HK2 modulates the proliferation and apoptosis of the HCC cells by NaBu was further investigated. The results of the CCK8 assay, Western blotting and flow cytometry showed that when HK2 was overexpressed, the inhibition of proliferation and the induction of apoptosis were weakened (Figure 3). These results suggest that HK2 inhibition is essential for the modulatory effects of NaBu on aerobic glycolysis, proliferation and apoptosis in the HCC cells. Our previous research showed that among the HCC‐LM3, Bel‐7402, SMMC‐7721, Huh 7, HepG2, LO2 and QSG‐7701 cell lines, HCC‐LM3 and Bel‐7402 exhibited the highest rate of aerobic glycolysis and the highest protein levels of HK2.^32^ In the present study, the results of the CCK8 assay indicated that HCC‐LM3 and Bel‐7402 cell lines were more sensitive to NaBu treatment than SMMC‐7721, HepG2 and LO2 cell lines. This finding indirectly demonstrates that the modulation of HK2 and glycolysis plays a key role in the anti‐HCC effects of NaBu.

C‐myc is a common oncogene that enhances aerobic glycolysis in the cancer cells by transcriptionally activating GLUT1, HK2, PKM2 and LDH‐A.^62^ NaBu suppresses the expression of c‐myc in HCC cells.^23^ In the present study, the mRNA expression of c‐myc was significantly inhibited in both HCC‐LM3 and Bel‐7402 cell lines by NaBu. The results of Western blotting showed that NaBu inhibited the expression of c‐myc in the total and nuclear lysate in a dose‐depedent manner. NaBu suppressed the expression of c‐myc in the tumour tissues. After transfection with c‐myc‐overexpressing lentivirus, the inhibitory effects of NaBu on the expressions of total c‐myc, nuclear c‐myc, and HK2 and lactate production and glucose uptake were attenuated. Furthermore, these effects were reversed by HK2 knockdown. In addition, when the c‐myc expression was ablated by siRNA transfection, 3 mM NaBu failed to modulate the protein expression of HK2 in the HCC cells, which implies that c‐myc was actually the target of NaBu when inhibiting HK2 expression (Figure 4). The above findings indicate that the modulatory effect of NaBu on the c‐myc pathway is essential for the inhibition of HK2 and aerobic glycolysis by NaBu in the HCC cells.

Sorafenib is the first‐line target drug to treat late‐stage HCC and prolong the survival of patients. However, few patients derive long‐term benefits from sorafenib owing to the early occurrence of sorafenib resistance.^63^ Many mechanisms underly sorafenib resistance in the HCC cells. Enhanced lactate production and glucose consumption and impaired mitochondrial OXPHOS have been observed in the HCC cells after sorafenib administration.^28^, ^30^ Key enzymes in the glycolytic pathway, including GLUT1, HK2, PFK1, PKM2 and LDH‐A, are overexpressed in sorafenib‐resistant HCC cell lines, which upregulates aerobic glycolysis.^9^, ^10^, ^64^ Inhibiting these enzymes has been reported to exhibit synergistic effects with sorafenib in treating HCC.^28^, ^65^ Lozano et al. documented that butyrate enhanced the sensitivity of cholangiocarcinoma cells to sorafenib by increasing sorafenib uptake.^66^ In the present study, aerobic glycolysis was enhanced and mitochondrial OXPHOS was impaired in the HCC‐LM3 cell line after sorafenib treatment, which was reversed by NaBu treatment. Accordingly, NaBu augmented the ability of sorafenib to inhibit proliferation and induce apoptosis in the HCC cells both *in vitro* and *in vivo* (Figure 5).

There are some limitations to our research. For instance, NaBu treatment suppressed the protein expressions of PFK1 and LDH‐A in HCC‐LM3 and Bel‐7402 cell lines *in vitro*. However, the results of IHC staining and Western blotting implied that 200 mg/kg NaBu did not affect the expression of PFK1 or LDH‐A in mouse tumour tissues. This discrepancy warrants further investigation.

## 5 CONCLUSION

Our findings suggest that NaBu, a gut microbiota metabolite, inhibits the expression of HK2 and subsequently downregulates aerobic glycolysis and cell proliferation, and induces apoptosis by suppressing the c‐myc pathway. NaBu impairs the enhancement of aerobic glycolysis in the HCC cells by sorafenib and improves the effect of the drug on HCC cells both *in vitro* and *in vivo*. The findings of the present study may aid us in better understanding the role of butyrate in hepatocellular carcinogenesis and the treatment of HCC.

## CONFLICT OF INTEREST

The authors declare no conflicts of interest.

## AUTHOR CONTRIBUTIONS

**Qiang Yu:**Conceptualization (lead); data curation (lead); formal analysis (lead); investigation (lead); methodology (lead); writing – original draft (lead). **Weiqi Dai:** Formal analysis (lead); writing – original draft (lead). **Jie Ji:** Writing – review & editing (supporting). **Liwei Wu:** Methodology (supporting). **Jiao Feng:** Methodology (supporting). **Jingjing Li:** Software (lead). **Yuanyuan Zheng:** Validation (supporting). **Yan Li:** Software (supporting). **Ziqi Cheng:** Investigation (supporting). **Jie Zhang:** Writing – review & editing (supporting). **Jianye Wu:** Resources (supporting). **Xunafu Xu:** Conceptualization (lead); writing – review & editing (lead). **Chuan‐yong Guo:** Conceptualization (lead); resources (lead); writing – review & editing (lead).

## Supporting information

Fig S1

Fig S2

Table S1‐S2

## Data Availability

The datasets generated during and/or analysed during the current study are available from the corresponding author on reasonable request.
