# Supplementary figures and images for: Sodium butyrate inhibits aerobic glycolysis of hepatocellular carcinoma cells via the c‐myc/hexokinase 2 pathway
Source: J Cell Mol Med. 2022 Apr 16;26(10):3031–45. doi: 10.1111/jcmm.17322 (PMC9097842; doi:10.1111/jcmm.17322)

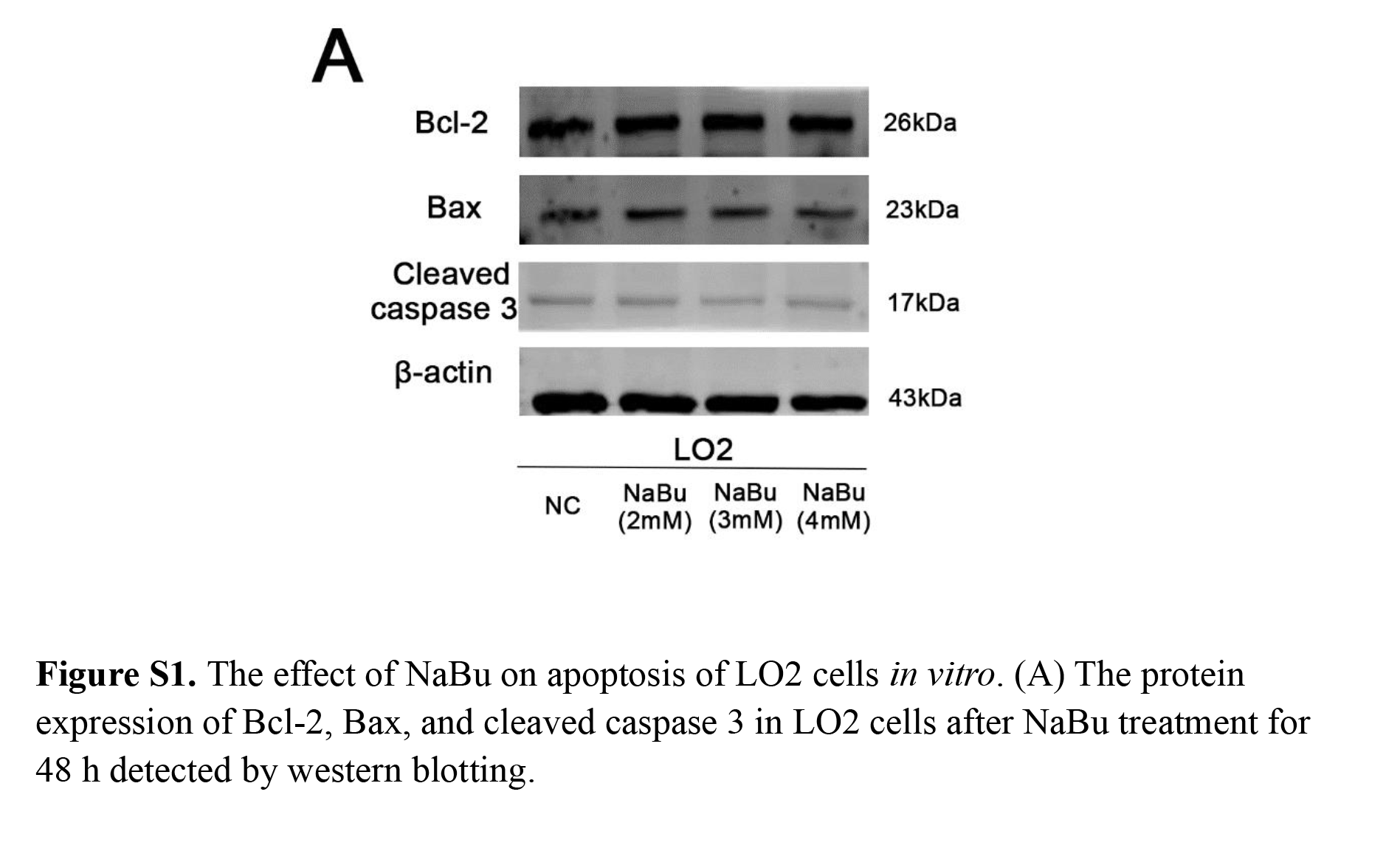

Supplement: Supplementary file 1 — Fig S1 [file JCMM-26-3031-s002.tif]

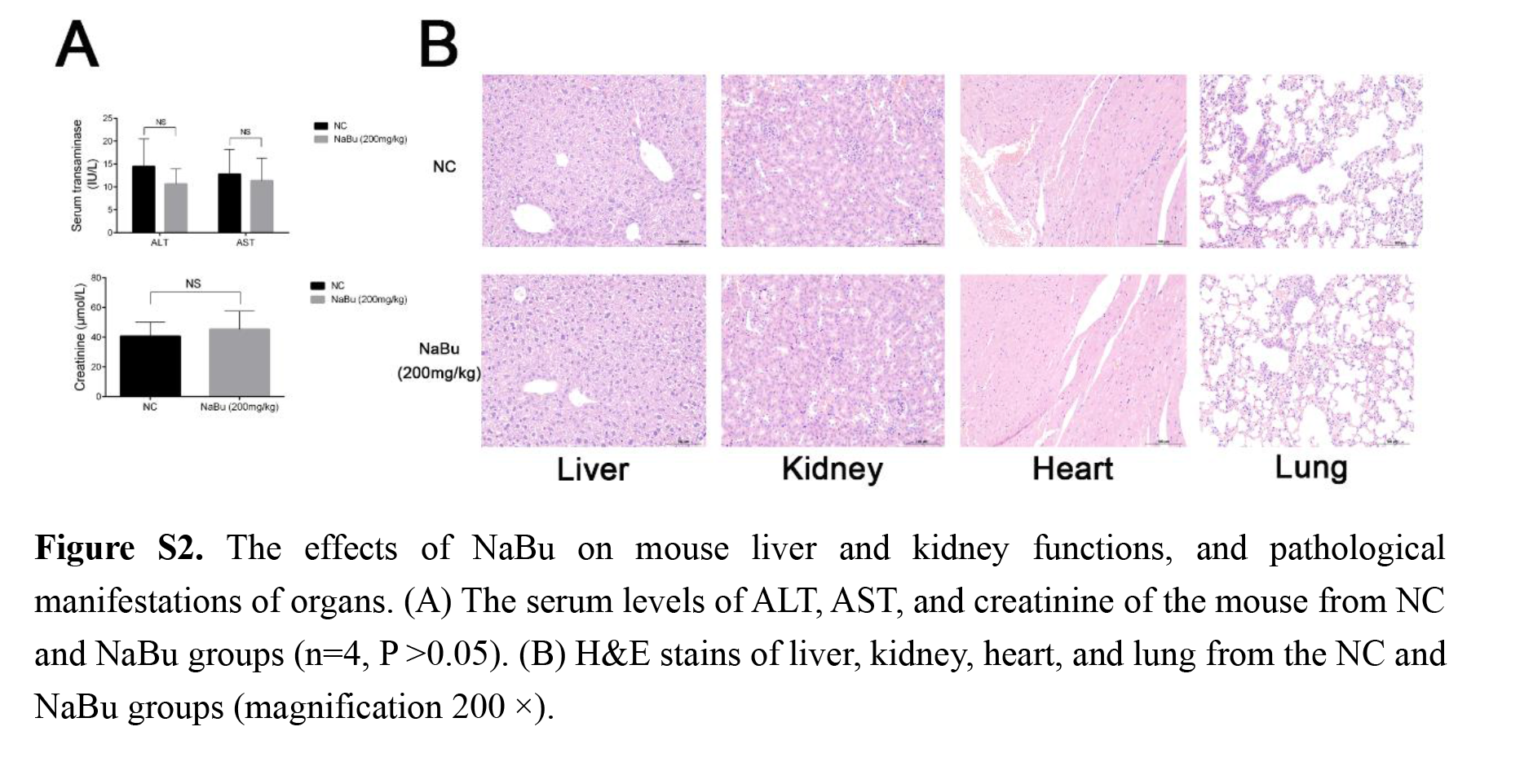

Supplement: Supplementary file 2 — Fig S2 [file JCMM-26-3031-s003.tif]
