# Supplementary material for: Sodium butyrate inhibits aerobic glycolysis of hepatocellular carcinoma cells via the c‐myc/hexokinase 2 pathway
Source: J Cell Mol Med. 2022 Apr 16;26(10):3031–45. doi: 10.1111/jcmm.17322 (PMC9097842; doi:10.1111/jcmm.17322)
Supplement: Supplementary file 3 — Table S1‐S2 [file JCMM-26-3031-s001.docx]

**Supplementary Table 1** The primary antibodies used in the study

| Antibody | Species | Dilution ratio | Supplier | Catalogue number |
| --- | --- | --- | --- | --- |
| β-actin | M | 1:1000 | CST | 3700 |
| PCNA | Rbt | 1:2000 | Proteintech | 10205-2-AP |
| Caspase 3 | Rbt | 1:1000 | Proteintech | 19677-1-AP |
| Caspase 9 | M | 1:1000 | Proteintech | 66169-1-Ig |
| Bax | Rbt | 1:1000 | Servicebio | GB11690 |
| Bcl-2 | Rbt | 1:1000 | Proteintech | 26593 |
| HK2 | Rbt | 1:1000 | Proteintech | 22029-1-AP |
| PFK1 | Rbt | 1:1000 | CST | 8175 |
| PKM2 | M | 1:2000 | Proteintech | 60268-1-Ig |
| LDH-A | Rbt | 1:1000 | CST | 3582 |
| OXPHOS | M | 1:1000 | Invitrogen | 45-8199 |
| c-myc | Rbt | 1:1000 | Epitomics | 1472-1 |
| Lamin B | Rbt | 1:1000 | Wanleibio | WL01775 |
| VDAC1 | Rbt | 1:1000 | Proteintech | 10866-1-AP |

Abbreviations for the table: M mouse; Rbt rabbit; CST Cell Signaling Technology (Danvers, MA, USA).

**Supplementary Table 2** Primers used for rt-PCR

| Gene name | Forward (5′-3′) | Reverse (5′-3′) |
| --- | --- | --- |
| β-actin | CTGGAACGGTGAAGGTGACA | AAGGGACTTCCTGTAACAATGCA |
| GLUT1 | GCCAGAAGGAGTCAGGTTCAA | TCCTCGGAAAGGAGTTAGATCC |
| HK2 | GAGCCACCACTCACCCTACT | CCAGGCATTCGGCAATGTG |
| PFKFB3 | TTGGCGTCCCCACAAAAGT | AGTTGTAGGAGCTGTACTGCTT |
| PFK1 | GTACCTGGCGCTGGTATCTG | CCTCTCACACATGAAGTTCTCC |
| PKM2 | ATGTCGAAGCCCCATAGTGAA | TGGGTGGTGAATCAATGTCCA |
| LDH-A | ATGGCAACTCTAAAGGATCAGC | CCAACCCCAACAACTGTAATCT |
| LDH-B | TGGTATGGCGTGTGCTATCAG | TTGGCGGTCACAGAATAATCTTT |
| HIF-1α | GAACGTCGAAAAGAAAAGTCTCG | CCTTATCAAGATGCGAACTCACA |
| c-myc | GTCAAGAGGCGAACACACAAC | TTGGACGGACAGGATGTATGC |
| AMPK-α | TTGAAACCTGAAAATGTCCTGCT | GGTGAGCCACAACTTGTTCTT |
| AMPK-β | CCACTCCGAGGAAATCAAGGC | CTGGGCGGGAGCTTTATCA |
| STAT3 | CAGCAGCTTGACACACGGTA | AAACACCAAAGTGGCATGTGA |
| PI3K | TATTTGGACTTTGCGACAAGACT | TCGAACGTACTGGTCTGGATAG |
| Akt1 | AGCGACGTGGCTATTGTGAAG | GCCATCATTCTTGAGGAGGAAGT |
| mTOR | GCAGATTTGCCAACTATCTTCGG | CAGCGGTAAAAGTGTCCCCTG |
